# Illustrating the Natural History of Influenza A Viruses through Art

**DOI:** 10.3201/eid2212.AC2212

**Published:** 2016-12

**Authors:** Robert G. Webster

**Affiliations:** St. Jude Children’s Research Hospital, Memphis, Tennessee, USA

**Keywords:** art science connection, emerging infectious diseases, art and medicine, about the cover, infectious diseases, Jenny Hammond, illustrating the natural history of influenza A viruses through art, The Natural History of Influenza A Viruses, respiratory disease, public health, influenza virus, viruses, influenza

**Figure:**
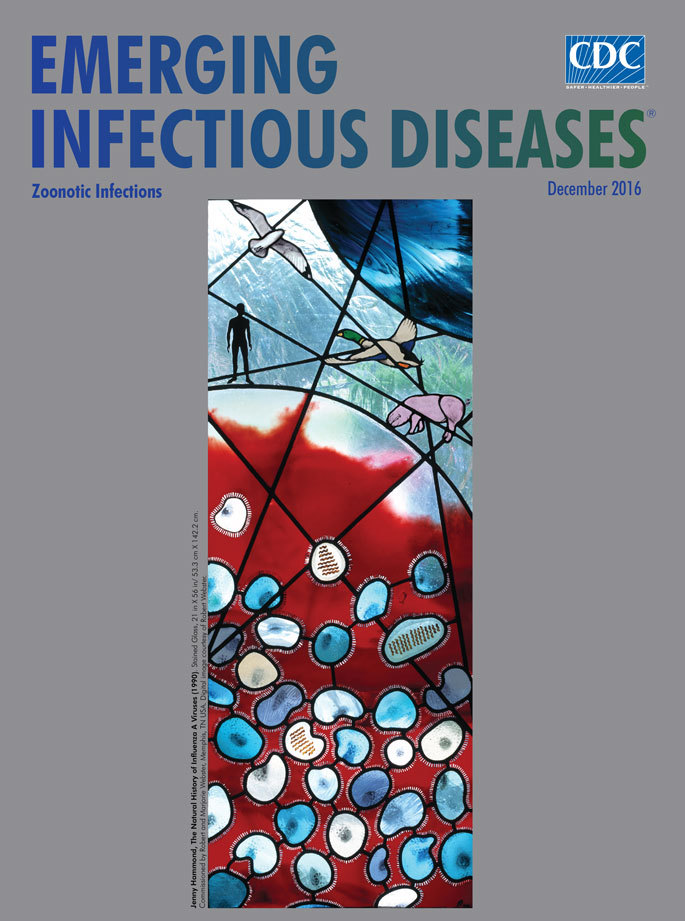
**Jenny Hammond, The Natural History of Influenza A Viruses (1990). Stained Glass, 21 in × 56 in / 53.3 cm × 142.2 cm.** Commissioned by Robert and Marjorie Webster, Memphis, TN, USA. Digital image courtesy of Robert Webster.

On a rainy and misty day in late 1989, my wife (Marjorie) and I were walking the path along the remnants of Hadrian’s Wall, a UNESCO world heritage site near the border between England and Scotland. The Romans began building this wall—which extends from the banks of the river Tyne on the west coast to Solvay Firth on the east coast of England—ostensibly to keep the “barbarian” Scots from plundering their English territory.

During lunch in a local pub, we discovered in a local publication a picture of a wonderful stained glass window depicting a dragon. We arranged to visit the artist, Jenny Hammond, at her nearby farm in Highgreenleycleugh, Northumberland, England, where we viewed her stained glass works firsthand and commissioned her to create a unique stained glass window that would detail the natural history of influenza. After we returned to our home in Memphis, Tennessee, we sent Hammond several review articles and electron micrographs to provide her some background on influenza A viruses. Hammond, in turn, shared her ideas through penciled sketches and over the next year completed the version that appears on this month’s EID journal cover (Figure).

**Figure:**
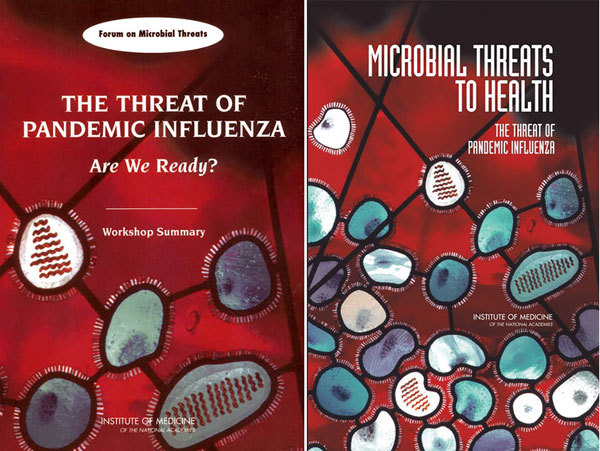
A different perspective of this stained glass window has also appeared on the cover of the book *Microbial Threats to Health: Emergence, Detection, and Response* (2003), by the Committee on Emerging Microbial Threats to Health in the 21st Century, Board on Global Health, Institute of Medicine, National Academies Press; another appears on the cover of a series of workshop summaries also published by the Institute of Medicine, National Academies Press.

After its transport by air, Marjorie and I installed the stained glass window into the premeasured window frame near the front door of our home. Visiting students and colleagues from around the world invariably ask to photograph the window. This stained glass window offers viewers a concise introduction to influenza in a One Health system in which viruses emerge from wild bird reservoirs and periodically cause pandemic diseases (such as influenza) in humans.

Although the term “One Health” was recently coined, it describes an ancient concept recognized by Hippocrates in his text “On Airs, Waters, and Places.” Scientists have noted the similarity in disease processes between animals and humans since the 1800s. Rudolf Virchow, a 19th century German pathologist and anthropologist, devised the term “zoonosis” to indicate web of the infectious diseases links between animals and humans, saying that “... between animal and human medicine there are no dividing lines—nor should there be.” In the 20th century, the One Health concept coalesced and gained momentum in the public health and animal health communities. The work of art depicted on this month’s cover depicts that interrelationship of human, animal, and environmental health.

The dark blue glass prominently positioned in the upper right signifies the global problem of influenza A viruses, which are associated with yearly epidemics and intermittent pandemics. The came strips, which provide structure for the window, also depict the spread of virus, which has a large reservoir and vast gene pool in wild migratory aquatic birds—including ducks and gulls represented in the window as well as shorebirds, geese, and terns. The influenza viruses can spread to pigs, considered the intermediate host, and to humans. The red background depicts the high fever in pigs and humans infected with the influenza virus.

Hammond also incorporated microscopic details essential to natural history of influenza A. Her depiction of the influenza virus particles show the spiky surface made up of the hemagglutinin that attaches the virus to the respiratory tract of the host and the neuraminidase that releases the virus from infected cells so that the virus can spread. She has depicted the RNA genome of 8 segments as separate threads. The particles with multiple threads illustrate how reassortment between influenza viruses gives rise to new pandemic strains.

Stained-glass windows have been appreciated for their utility and splendor for more than 1,000 years, and this engaging work of art reminds us that influenza A viruses—which can be easily spread between animals and human, use various host species, and exist in many different environments—remain an enduring and global health concern.
